# Supplementary material for: EXOSC10 is required for RPA assembly and controlled DNA end resection at DNA double-strand breaks
Source: Nat Commun. 2019 May 13;10:2135. doi: 10.1038/s41467-019-10153-9 (PMC6513946; doi:10.1038/s41467-019-10153-9)
Supplement: Supplementary file 1 — Supplementary Information [file 41467_2019_10153_MOESM1_ESM.pdf]

EXOSC10 is required for RPA assembly and controlled DNA end resection at DNA double-strand breaks

Domingo-Prim et al.

Supplementary Information

Supplementary Figure 1
Depletion of exosome catalytic subunits and effects on cell cycle progression

Supplementary Figure 2
EXOSC10 depletion inhibits the assembly of RPA foci

Supplementary Figure 3
Analysis of DNA end resection in U2OS cells

Supplementary Figure 4
Analysis of RNA levels and cleavage efficiency at site-specific DSBs

Supplementary Figure 5
DRIP-qPCR experiments showing RNaseH controls.

Supplementary Figure 6
ChIP analysis of RNAPII and TFIIS at I-PpoI-induced DSBs

Supplementary Figure 7
Expression of catalytically inactive EXOSC10 mutants in HeLa cells

Supplementary Figure 8
A catalytically inactive EXOSC10 mutant is recruited to sites of DNA damage

Supplementary Figure 9
SMART experiments in cells treated with transcription inhibitors

Supplementary Figure 10
Analysis of RNA levels at an intergenic DSB in DiVA cells

Supplementary Table 1
Sequences of the primers used in this study

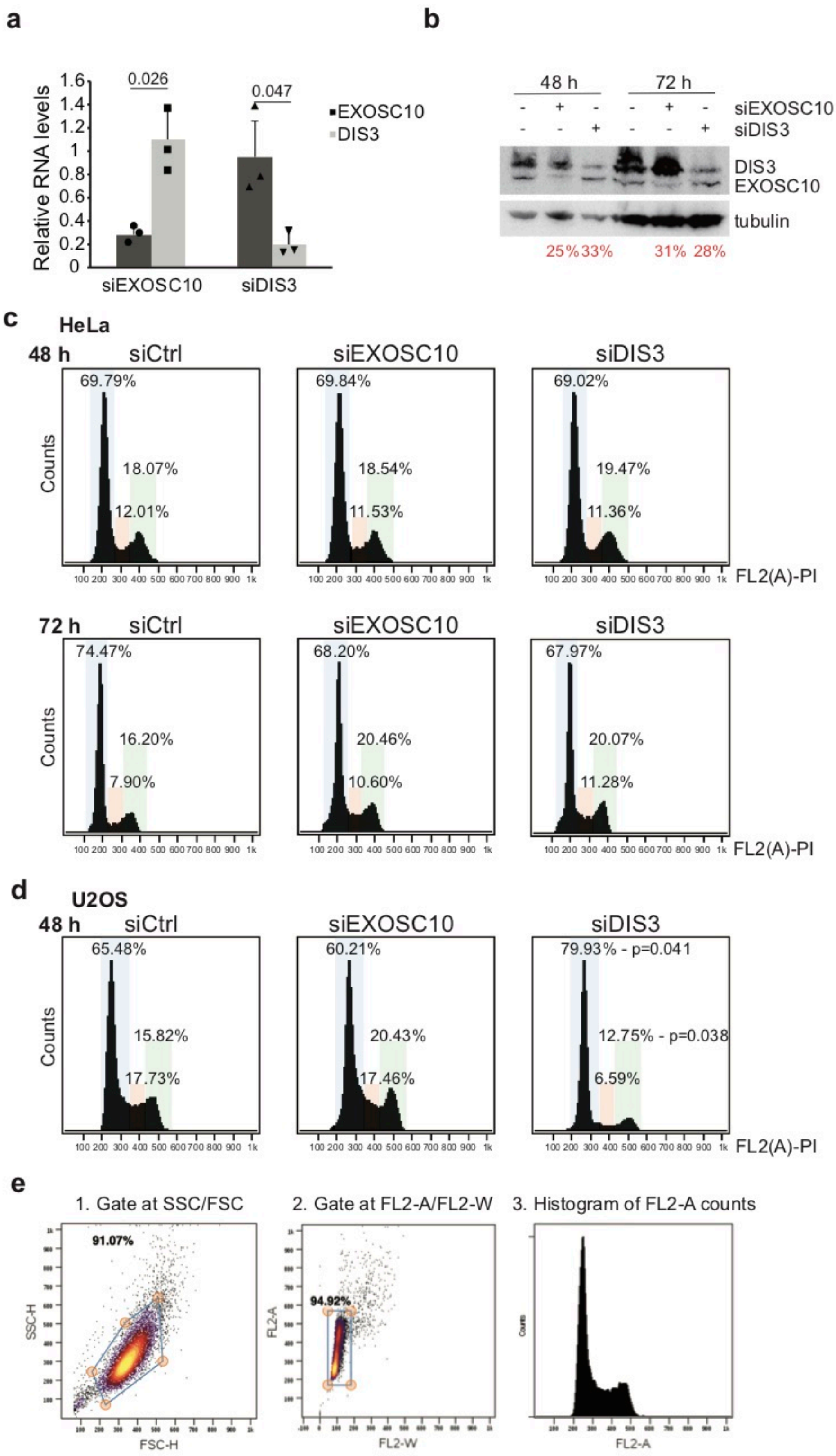

Supplementary Figure 1. Depletion of exosome catalytic subunits and effects on cell cycle progression

a) The graph shows relative levels of EXOSC10 and DIS3 mRNAs analysed by RT-qPCR in HeLa cells that were treated with siRNA for each of the exosome subunits for 48 h. ARRP mRNA levels were quantified in parallel for normalization. The values are averages and the error bars are s.d. (n= 3 independent experiments). Statistical testing

was done using a one-tail Student's t-test and significant p-values are shown in the figure. **b)** Western blot showing the depleted protein levels of EXOSC10 and DIS3. Tubulin was used as a loading control. The levels of EXOSC10 and DIS3 in siRNA-treated cells were normalized to tubulin and compared to the levels in control cells. Source data are provided as a Source Data file. **c-d)** The plots show the effect of EXOSC10 and DIS3 depletion on the cell cycle of HeLa cells and DiVA U2OS cells. The cells were stained with propidium iodide and analysed by flow cytometry. The results are expressed as a percentage of counts at FL-A. 10^4 cells were analysed in each condition. **e)** The figure exemplifies the gating strategy. Source data are provided as a Source Data file.

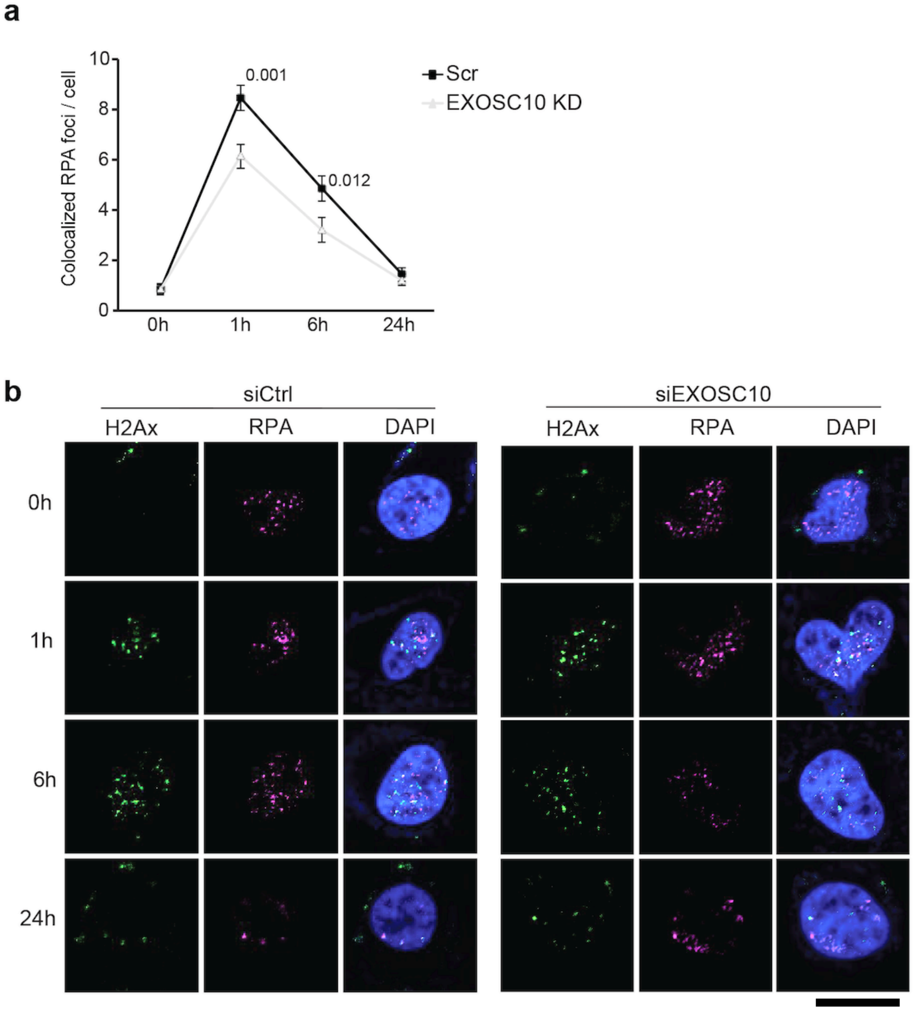

Supplementary Figure 2. EXOSC10 depletion inhibits the assembly of RPA foci in cells exposed to ionizing radiation

HeLa cells were transfected either with siCtrl or siEXOSC10, irradiated 48 h after transfection with ionizing radiation (5 Gy), and fixed at different time-points as indicated. The cells were stained with antibodies against γH2AX and RPA. **a)** The plot shows the average number of RPA foci that colocalize with γH2AX foci per cell. The statistical significance of the changes observed between EXOSC10-depleted and control samples was tested using a Mann-Whitney's test and significant p-values are shown in the figure (n=40 cells analysed for each time point, from 2 independent experiments). The error bars represent s.e.m. Source data are provided as a Source Data file. **b)** Examples of the immunostaining are provided. The scale bar represents 20 μm.

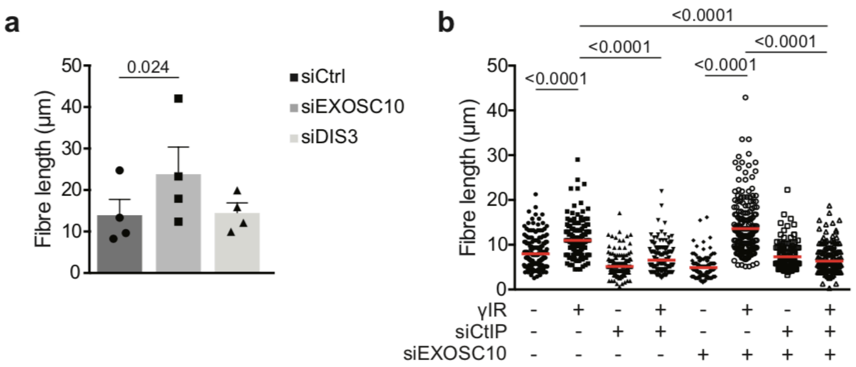

Supplementary Figure 3: Analysis of DNA end resection in U2OS cells

a) The graph shows the average fibre length measured by SMART in controls cells (siCtrl) and in cells depleted of EXOSC10 or DIS3. The error bars represent s.e.m. of three independent experiments. P-values were calculated with a paired Student t-test (n=4 independent experiments). **b)** U2OS cells were depleted for either CtIP, EXOSC10 or both and treated with or without γ-radiation (10 Gy). The graph shows the fibre length quantified by SMART (n=200 fibres analysed). P-values were calculated with Mann-Whitney's test (n=200). Source data are provided as a Source Data file.

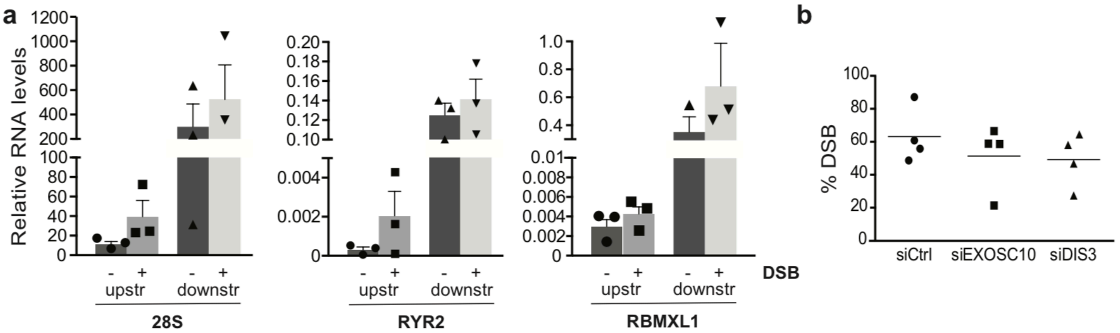

Supplementary Figure 4. Analysis of RNA levels and cleavage efficiency at site-specific DSBs

a) RNA levels were measured by ssRT-qPCR at 28S and RYR2 I-PpoI sites and at RMBXL1 AsiSI site. For the analysis of I-PpoI DSBs, HeLa cells were transfected with pOPRSVI/MCS-I-PpoI and RNA was extracted 20 h after the transfection. For the analysis of the AsiSI DSB, DiVA cells were treated with 4-OHT for 4 h. The graph shows the relative RNA levels normalized to ARPP before and after inducing DNA damage. The differences between –DSB and +DSB were tested with a paired Student's t-test (n=3 independent experiments). The error bars represent s.e.m. **b)** HeLa cells were knocked-down for EXOSC10 or DIS3 for 24 h and transfected with pOPRSVI/MCS-I-PpoI. Genomic DNA was extracted 20 h after the transfection and the percentage of DSBs produced by I-PpoI was analysed by qPCR using PCR primers located at each side of the DSB in the 28S locus. The plot shows the percentage of DSBs produced in the different knock-down conditions (n=4 independent experiments). Source data are provided as a Source Data file.

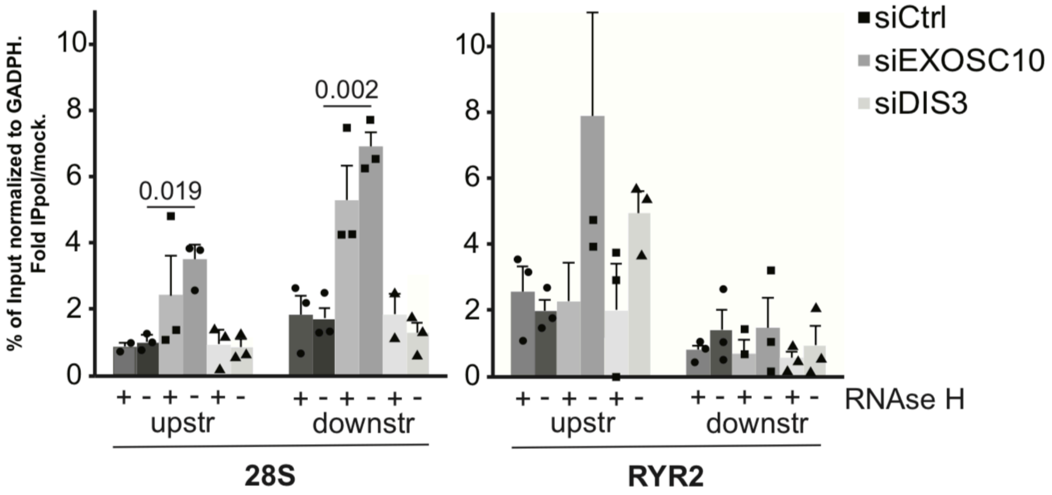

Supplementary Figure 5. DRIP-qPCR experiments showing RNaseH controls

DRIP-qPCR was performed in HeLa cells 20 h after transfection with the pOPRSVI/MCS-I-PpoI plasmid. Negative control samples were also digested with 10 U RNase H1 before immunoprecipitation. The graph shows the relative DRIP-qPCR levels in cells treated with either siEXOSC10 or siDIS3 compared to control cells (siCtrl). DRIP-qPCR levels were normalized to GADPH. Error bars show s.e.m. of three independent experiments. Statistical testing was carried out with a paired Student's t-test.
Source data are provided as a Source Data file.

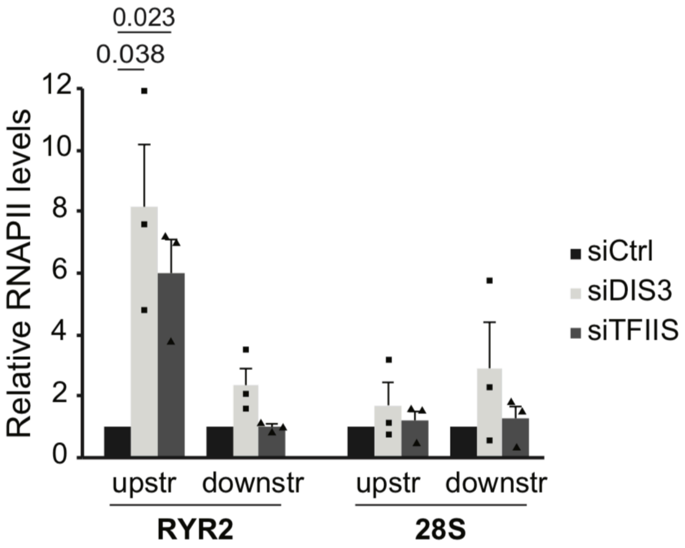

Supplementary Figure 6. ChIP analysis of RNAPII at I-PpoI-induced DSBs

RNAPII ChIP-qPCR was performed in HeLa cells depleted of either DIS3 or TFIIS. The graph shows the fold change of the percentage of input relative to the control. ChIP-qPCR values were normalized to ARPP. The error bars represent s.e.m. of three independent experiments. P-values were calculated with a one-sample Student t-test (n=3). Source data are provided as a Source Data file.

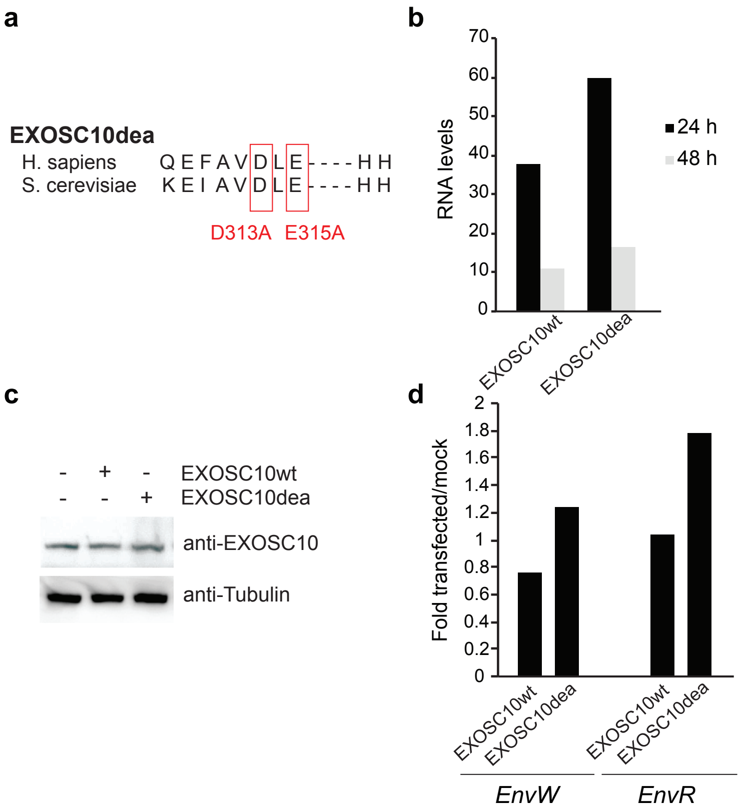

Supplementary Figure 7. Expression of a catalytically inactive EXOSC10 mutant in HeLa cells

The pOPRSVI-EXOSC10 plasmid was used to produce site-specific mutations and to express the mutated protein in HeLa cells. **a)** The figure shows the mutations in the conserved sequences of EXOSC10 and its Rrp6p ortholog. **b)** The expression of EXOSC10 wild-type and mutant was measured by RT-qPCR using primers that distinguish endogenous from recombinant transcripts. The plot shows the relative mRNA levels of the recombinant mRNA compared to the levels of endogenous EXOSC10 mRNA analysed 24 or 48 h after transfection. Data from one experiment. **c)** Western blotting was used to visualize the mutant protein levels. **d)** The effect of the expression of mutant EXOSC10 on the levels of two known RNA targets, EnvW and EnvR, was analysed by RT-qPCR. Total RNA was extracted 24 h after pOPRSVI-EXOSC10 mutant transfection. ARPP mRNA was used for normalization. Data from one experiment.
Source data are provided as a Source Data file.

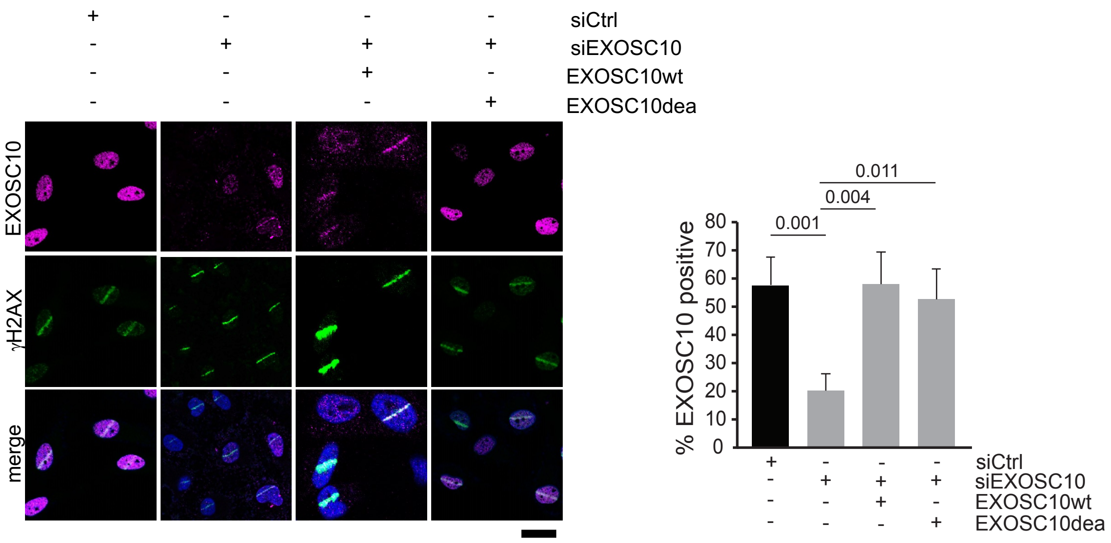

Supplementary Figure 8. A catalytically inactive EXOSC10 mutant is recruited to sites of DNA damage

HeLa cells were transfected with siEXOSC10 or siCtrl and transfected again 24 h later with either pOPRSVI-EXOSC10^{wt} or pOPRSVI-EXOSC10^{dea}, as indicated. The cells were treated with a low concentration of Actinomycin D (40 nM) 24 h after the second transfection to facilitate the imaging of irradiated stripes, as in Figure 1a, laser micro-irradiated and immunostained with antibodies against EXOSC10 and γH2AX. The bar plot in the right part of the figure shows the percentage of γH2AX-positive stripes that were co-stained with antibodies against EXOSC10 in each condition. Statistical testing was done using a Mann-Whitney's test and significant p-values are shown in the plot (n>20 stripes analysed in each condition, from two independent experiments). The error bars represent s.e.m. The scale bar represents 20 μm.

Source data are provided as a Source Data file.

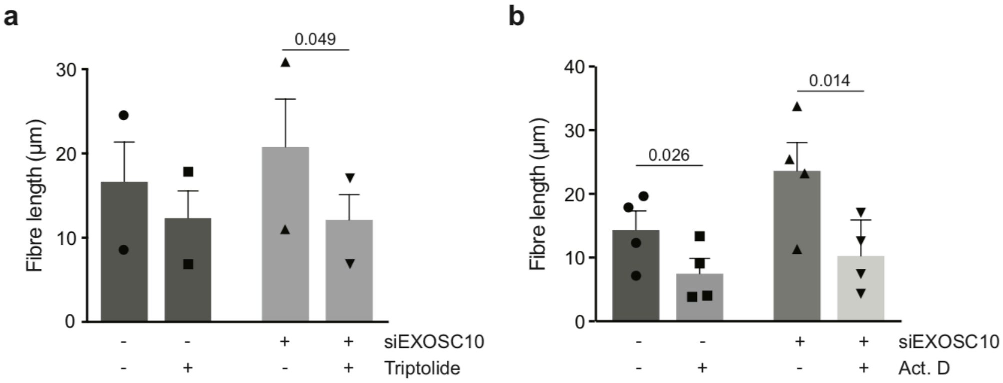

Supplementary Figure 9. SMART experiments in cells treated with transcription inhibitors

SMART experiments in control cells and EXOSC10-depleted cells were carried out as described in Figure 6e-f. The cells were treated with Triptolide (**a**) or 8 µM ActD 10 µM (**b**) as described. The plots show averages of the median values from a number of independent experiments (n= 2 and 4 independent experiments in **a** and **b**, respectively). Error bars show s.e.m. Statistical testing was done using a Student's t-test and significant p-values are shown in the plot. Source data are provided as a Source Data file.

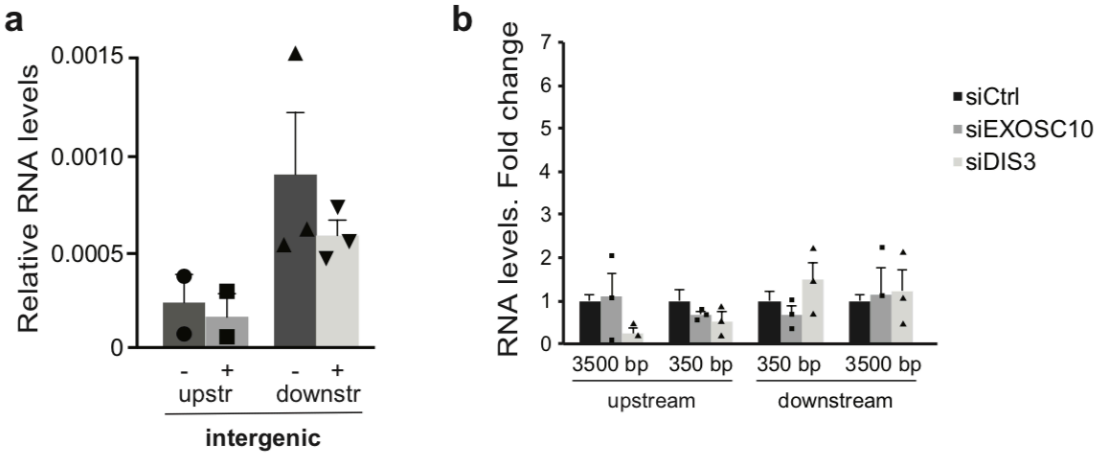

Supplementary Figure 10. Analysis of RNA levels at an intergenic DSB in DiVA cells

a) RNA levels were measured by ssRT-qPCR at a AsiSI site located at chr13:105238551. DiVA cells were treated with 4-OHT for 4 h. The graph shows the relative RNA levels normalized to ARPP before (–) and after (+) inducing DNA damage. Note that RNA levels at this locus are at least two orders of magnitude lower than those measured at other DSBs (compare this figure with Supplementary Figure 4a, locus RBMXL1). n=3 independent experiments. **b)** The graph shows the fold change of RNA levels in cells transfected with siEXOSC10 or siDIS3 compared to the levels in control cells (siCtrl). RNA levels were normalized to ARPP. Depletion of exosome subunits did not reveal dilncRNAs in this locus. n=3 independent experiments. Error bars show s.e.m. Source data are provided as a Source Data file.

Supplementary Table 1. Sequences of the primers used in this study

Primer		Sequence
ARPP	F	GCACTGGAAGTCCAACTACTTC
	R	TGAGGTCCTCCTTGGTGAACAC
GADPH	F	CCTGCTTCACCACCTTCTTGATGTC
	R	CAAGGTCATCCCAGAGCTGAACG
EXOSC10	F	CATGCCATTTCATATCCTCCC
	R	GATGAATTTCTTGAGGCAGATGT
DIS3	F	GCACAGTGCCCCATGAAATTGCCCCA
	R	AGCCACCGTGCCTGGCCTGCT
AsiSI_DSB 350bp up	F	GAATCGGATGTATGCGACTGATC
	R	TTCCAAAGTTATTCCAACCCGAT
AsiSI_DSB 1500bp up	F	TGAGGAGGTGACATTAGAACTCAGA
	R	AGGACTCACTTACACGGCCTTT
AsiSI_DSB 3500bp up	F	TCCTAGCCAGATAATAATAGCTATACAAACA
	R	TGAATAGACAGACAACAGATAAATGAGACA
AsiSI_DSB No DSB A	F	ATTGGGTATCTGCGTCTAGTGAGG
	R	GACTCAATTACATCCCTGCAGCT
AsiSI_DSB No DSB B	F	CACACTCAGCAAACTCGGAAT
	R	GGAAGCTTTTACTCTTCCACCA
AsiSI_DSB Across	F	GATGTGGCCAGGGATTGG
	R	CACTCAAGCCCAACCCGT
AsiSI_DSB 350bp dw	F	GGATGGTCTCGATCTCCTGA
	R	GAAAGAGGCTCACCCCTGTA
AsiSI_DSB 3500bp dw	F	GGGAGATTTCACCCCAGACT
	R	GGCAGCTCCCTAAGAAGGAA
AsiSI_Intergenic_DSB 350bp up	F	AAAATCTGAGCTTCCGGTGA
	R	TTCAATTTCCATTCATTAATTTGG
AsiSI_Intergenic_DSB 1500bp up	F	GCTAACACAAGCCCATTGAGA
	R	CCAACAATTGCTTAGATGTTTTAGTG
AsiSI_Intergenic_DSB 3500bp up	F	AAGAGGGCACCACCAGAAC
	R	TTCCCCCAGGGTCAAAATA
AsiSI_Intergenic_DSB Across	F	GACTGGTGGAAGACACGTCA
	R	AGCAGACACCACCATCCTTC
RYR2 up	F	GGATATAGCATTGATTTATTCTGAAA
	R	CCCTTGTGACCTGAAAGAGA
RYR2 dw	F	TCAAGACCCATCAGTGCTGT
	R	AATCCCTGCCTTTGTTTTCC
28S up	F	GGGGGAGAGGGTGTAAATCT
	R	TTGCCGACTTCCCTTACCTA
28S dw	F	CGATGTCGGCTCTTCCTATC
	R	AACCTGTCTCACGACGGTCT
