# Supplementary material for: EXOSC10 is required for RPA assembly and controlled DNA end resection at DNA double-strand breaks
Source: Nat Commun. 2019 May 13;10:2135. doi: 10.1038/s41467-019-10153-9 (PMC6513946; doi:10.1038/s41467-019-10153-9)
Supplement: Supplementary file 3 — Reporting Summary [file 41467_2019_10153_MOESM3_ESM.pdf]

nature research

Corresponding author(s): Neus Visa

Last updated by author(s): Apr 5, 2019

Reporting Summary

Nature Research wishes to improve the reproducibility of the work that we publish. This form provides structure for consistency and transparency in reporting. For further information on Nature Research policies, see Authors & Referees and the Editorial Policy Checklist.

Statistics

For all statistical analyses, confirm that the following items are present in the figure legend, table legend, main text, or Methods section.

n/a	Confirmed	
☐	☒	The exact sample size (*n*) for each experimental group/condition, given as a discrete number and unit of measurement
☐	☒	A statement on whether measurements were taken from distinct samples or whether the same sample was measured repeatedly
☐	☒	The statistical test(s) used AND whether they are one- or two-sided<br>*Only common tests should be described solely by name; describe more complex techniques in the Methods section.*
☒	☐	A description of all covariates tested
☐	☒	A description of any assumptions or corrections, such as tests of normality and adjustment for multiple comparisons
☐	☒	A full description of the statistical parameters including central tendency (e.g. means) or other basic estimates (e.g. regression coefficient) AND variation (e.g. standard deviation) or associated estimates of uncertainty (e.g. confidence intervals)
☒	☐	For null hypothesis testing, the test statistic (e.g. *F*, *t*, *r*) with confidence intervals, effect sizes, degrees of freedom and *P* value noted<br>*Give P values as exact values whenever suitable.*
☒	☐	For Bayesian analysis, information on the choice of priors and Markov chain Monte Carlo settings
☒	☐	For hierarchical and complex designs, identification of the appropriate level for tests and full reporting of outcomes
☒	☐	Estimates of effect sizes (e.g. Cohen's *d*, Pearson's *r*), indicating how they were calculated

Our web collection on statistics for biologists contains articles on many of the points above.

Software and code

Policy information about availability of computer code

Data collection	The following systems were used for data collection:<br>• Confocal microscopy: LSM 780 system with ZEN black 2011, service pack 7, Carl Zeiss.<br>• Quantitative PCR: Rotor-Gene Q series software 1.7.<br>• SMART: Eclipse NI-E, Nikon, using IS ELEMENTS Nikon software.<br>• Small RNA-seq: BaseSpace version 5.1, Illumina.
Data analysis	Excel, Fiji, GraphPad Prism, FlowJo BD Biosciences for flow cytometry, Adobe Photoshop CS4 for fiber length measurements, R software package v3.2.2 and R studio version 1.1.4 for RNA-seq data analysis.

For manuscripts utilizing custom algorithms or software that are central to the research but not yet described in published literature, software must be made available to editors/reviewers. We strongly encourage code deposition in a community repository (e.g. GitHub). See the Nature Research guidelines for submitting code & software for further information.

Data

Policy information about availability of data

All manuscripts must include a data availability statement. This statement should provide the following information, where applicable:

- Accession codes, unique identifiers, or web links for publicly available datasets
- A list of figures that have associated raw data
- A description of any restrictions on data availability

The small RNA-seq data are available in the NCBI Gene Expression Omnibus (GEO) under the accession number GSE113109. Custom scripts used for the small RNA seq analysis are available from the authors upon request. The source data underlaying Figures 1b-f, 2a-c, 3a-c, 4b-d, 5a-c, 6a-f, 7a-c and Supplementary Figures 1-10 are provided as a Source Data file.

Field-specific reporting

Please select the one below that is the best fit for your research. If you are not sure, read the appropriate sections before making your selection.

☒ Life sciences ☐ Behavioural & social sciences ☐ Ecological, evolutionary & environmental sciences

For a reference copy of the document with all sections, see nature.com/documents/nr-reporting-summary-flat.pdf

Life sciences study design

All studies must disclose on these points even when the disclosure is negative.

Sample size	For biochemical assays, at least three independent experiments were carried out. For microscopy assays (laser confocal microscopy and SMART imaging), the number of cells or tracks analyzed was based on previous experience and was between 50 and 150. The number of independent experiments or number of cells analyzed are indicated in the figure legends.
Data exclusions	No data were excluded from the analyses.
Replication	Attempts to replicate were successful.
Randomization	n/a
Blinding	Investigators were not blinded.

Reporting for specific materials, systems and methods

We require information from authors about some types of materials, experimental systems and methods used in many studies. Here, indicate whether each material, system or method listed is relevant to your study. If you are not sure if a list item applies to your research, read the appropriate section before selecting a response.

Materials & experimental systems

n/a	Involved in the study
☐	☒ Antibodies
☐	☒ Eukaryotic cell lines
☒	☐ Palaeontology
☒	☐ Animals and other organisms
☒	☐ Human research participants
☒	☐ Clinical data

Methods

n/a	Involved in the study
☒	☐ ChIP-seq
☐	☒ Flow cytometry
☒	☐ MRI-based neuroimaging

Antibodies

Antibodies used	The primary antibodies used for Western blotting and immunofluorescence were: mouse anti-EXOSC10 (sc-374595, Santa Cruz Biotechnology), rabbit anti-EXOSC10 (ab50558), rabbit anti-DIS3 (ab176802, Abcam), mouse anti-Tubulin (Sigma, T5168), rabbit anti-γH2AX (#9718, Cell Signaling Technology), mouse anti-γH2AX (ab26350, Abcam), rabbit anti-RAD51 (ab63801, Abcam), mouse anti-RPA (MABE285, Merck Millipore), mouse anti-CtIP (61141, Active Motif), rat anti-BrdU (B8434, Sigma-Aldrich) and mouse monoclonal anti-BrdU (RPN20AB, Sigma). Fluorophore-conjugated secondary antibodies used for immunofluorescence were: goat anti-mouse Alexa594 (115-585-003, Jackson Immunoresearch), donkey anti-rabbit-FITC (711-096-152, Jackson Immunoresearch), goat anti-rat Alexa647 (112-605-143, Jackson Immunoresearch). Secondary antibodies for Western blotting were: HRP-conjugated goat anti-mouse (P0447, Dako) and HRP-conjugated goat anti-rabbit (P0448, Dako).
Validation	Anti EXOSC10, DIS3, Tubulin, and RPA detected bands of the expected size. The other antibodies were not validated.

Eukaryotic cell lines

Policy information about cell lines

Cell line source(s)	HeLa cells (ECACC, 93021013) were purchased from Sigma Aldrich. Diva cells were provided by Gaelle Legube (University of Toulouse, France). U2OS-DR-GFP and U2OS-EJ5-GFP we provided by the Huertas laboratory. The U2OS-DR-GFP cell line is originally from Stephen P. Jackson laboratory (Sartori et al., Nature 450, 509-514, 2007). The U2OS-EJ5-GFP cell line is from Jimeno et al., Nucleic Acids Res. 43, 987-999, 2015.
Authentication	None of the cell lines were authenticated.

Mycoplasma contamination	Cells were negative for mycoplasma contamination.
Commonly misidentified lines (See ICLAC register)	n/a

Flow Cytometry

Plots

Confirm that:

☒ The axis labels state the marker and fluorochrome used (e.g. CD4-FITC).

☒ The axis scales are clearly visible. Include numbers along axes only for bottom left plot of group (a 'group' is an analysis of identical markers).

☒ All plots are contour plots with outliers or pseudocolor plots.

☒ A numerical value for number of cells or percentage (with statistics) is provided.

Methodology

Sample preparation	Experimental procedures are provided in the Methods section.
Instrument	A FACSCalibur (BD Biosciences) was used for flow cytometry.
Software	FlowJo software (BD Biosciences).
Cell population abundance	n/a
Gating strategy	SSC/FSC (y/x) plots were used to remove cell debris. For cell cycle analyses, FL2-A/FL2-W plots were used to gate single cell population. The figures show percentage of cells in G1, S and G2-M based on counts/FL2-A.

☒ Tick this box to confirm that a figure exemplifying the gating strategy is provided in the Supplementary Information.
